# Living in the Dark: Exploring the Factors Driving Nocturnal Activity in Three Lemur Species

**DOI:** 10.1002/ece3.72819

**Published:** 2026-02-19

**Authors:** Hasinavalona Rakotoarisoa, Jadelys Tonos, Hannah Hilden‐Reid, Onja H. Razafindratsima

**Affiliations:** ^1^ Department of Integrative Biology University of California Berkeley Berkeley California USA; ^2^ Mention Zoologie et Biodiversité Animale, Université d'Antananarivo Antananarivo Madagascar; ^3^ Department of Biology San Francisco State University San Francisco California USA

**Keywords:** animal behavior, cathemerality, movement, primates, temporal adaptation, tropical forests

## Abstract

Many animal species have adapted to being active both during the day and night (i.e., cathemeral) as an ecological response to the varying environmental conditions that occur along the day‐night cycle. Understanding the extent of nocturnal activities of cathemeral species and the factors that may influence such patterns can, thus, provide critical insights into the mechanisms shaping animal behavior and evolutionary adaptation. With a focus on three lemur species ( *Eulemur rubriventer* , *E. rufifrons* , and *Varecia variegata editorum* ) in a Malagasy tropical rainforest, we investigated how nocturnal luminosity (NLI), temperature, and rainfall influenced their active state and distance traveled at night compared to the day. We monitored them across 24‐h cycles from May 2023 to September 2024 using GPS collars. We found that both *Eulemur* species displayed distinct cathemeral activity patterns with longer distances traveled at nighttime than during the day; however, there was no difference in range sizes between day and night for *E. rufifrons* . *Varecia variegata editorum* individuals displayed fewer active states at night, shorter nocturnal travel distances, and a smaller nighttime range than during the day. We also found that NLI, temperature, and rainfall influenced the amount of nocturnal activity and the distances traveled at night, but the influencing factors varied across the three species. These findings highlight the dynamic nature of lemur activity patterns, highlighting the potential adaptive role of cathemerality in response to changing environments. They also point out the critical role of astronomical variations (i.e., moonlight conditions) and climatic variables in shaping animal ecology. More broadly, our findings provide important insights for the conservation planning of endangered species, as nocturnal activity patterns may influence their vulnerability to nighttime hunting, habitat disturbance, and the availability of light penetrating the canopy, especially as environmental changes and habitat loss continue to threaten animal survival.

## Graphical abstract

We investigated the factors driving nocturnal activities of three predominantly diurnal primate species in a Malagasy tropical rainforest, focusing on their active state, distance traveled, and home range. Our results highlight the critical role of astronomical variations (i.e., moonlight conditions) and climatic variables in shaping animal ecology. More broadly, our findings provide important information for the conservation planning of endangered species that may be vulnerable to changing environmental and light conditions, especially as climate change and habitat loss continue to threaten animal survival.

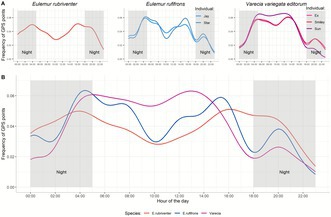

## FINTINA

Betsaka amin'ireo karazana biby no manova ny fomba fiainany ho lasa mandeha amin'ny atoandro sy amin'ny alina manoloana ny fiovaovan'ny tontolo iainana ao anatin'ny andro iray. Ny fandinihina ny fivelaran'ny hetsika ataon'izy ireo amin'ny alina, sy ireo antony mety hitarika an'izany fiovana hetsika izany, dia manampy hahafantarana ireo toe‐javatra mamolavola ny fitondran‐tenan'ny biby, ny fomba fiainany mifanaraka amin'ny fivoarana misy, ary ny fomba hihatrehan'ny biby ireo sedra izay ateraky ny fiovana misy eo amin'ny tontolo iainana mandritra ny andro sy ny alina. Ny tanjon'ity fikarohana ity dia ny mamakafaka ireo antony mahatonga ny varika ho lasa mandeha amin'ny alina na dia mandeha amin'ny atoandro koa ary. Karazana varika telo no nimasohana: varimoty ( *Eulemur rubriventer* ), varika mavo ( *Eulemur rufifrons* ), ary varijatsy ( *Varecia variegata editorum* ) ao anatin'ny ala mandon'i Ranomafana. Nojerena tamin'izany ny fiantraikan'ny hazavana amin'ny alina (NLI), ny mari‐pana ary ny rotsakorana amin'ny hetsika ataon'izy ireo sy ny halavirana vitany amin'ny alina raha oharina amin'ny atoandro. Mba ahafahana mahazo ny tahirikevitra ilaina amin'ity fikarohana ity dia nasiana fitaovana afaka mandray ny toerana misy azy isan'andro ny biby tsirairay, nanomboka tamin'ny May 2023 ka hatramin'ny Septambra 2024. Mampiseho ny tahirikevitra fa mihetsika amin'ny alina avokoa ireo karazana varika telo ireo, saingy amin'ny ambaratonga samihafa. Ho an'ny varimoty sy varika mavo dia mitovitovy ny hetsika ataony amin'ny atoandro sy amin'ny alina, ary mahavita mandeha lavitra kokoa izy amin'ny alina. Fa ho an'ny varika mavo manokana anefa dia tsy misy fiovana ny habehan'ny faritra misy azy na amin'ny alina na amin'ny atoandro. Ho an'ny varijatsy kosa dia vitsy kokoa ny fotoana nihetsehany amin'ny alina, fohy kokoa ny halaviran‐dalana vitany, ary kely kokoa ny faritra misy azy amin'ny alina raha oharina amin'ny atoandro. Nahita ihany koa izahay fa ny hazavan'ny alina, ny mari‐pana ary ny rotsakorana dia misy fiantraikany amin'ny hetsika sy halaviran‐dalana vitan'ny varika amin'ny alina. Tsy mitovy anefa izany fiantraikany izany amin'ireo karazana varika telo. Ireo vokam‐pikarohana ireo dia manasongadina ny fiovan'ny fihetsika sy fitondran‐tenan'ny varika ary ny fomba fiainany mifanaraka amin'ny fiovana misy. Eo ihany koa ny anjara asan'ny fiovaovan'ny andro, ny hazavan'ny volana sy ireo singa amin'ny tontolo iainana amin'ny famolavolana ny fomba fiainan'ny biby. Amin'ny ankapobeny, ireo vokam‐pikarohana ireo dia afaka ampiasaina amin'ny fandrindrana ny fiarovana ireo karazam‐biby tandindomin‐doza. Mety misy fiantraikany amin'ny faharefon'izy ireo manoloana ny tahan'ny fihazana amin'ny alina, ny fikorontan'ny toeram‐ponenany, ary ny tahan'ny hazavana miditra ao anaty ala mantsy ny fomba fiainany sy ny hetsika ataony amin'ny alina, indrindra manoloana ny fiovaovan'ny toetr'andro sy ny fahapotehan'ny fonenan'izy ireo amin'izao, izay mbola mitera‐doza ho an'ny fiveloman'ny biby.

## 1 Introduction

Animal behavior has been subject to variable environmental conditions following daily, annual, seasonal, and even lunar periodicities (Bennie et al. 2014; Halle and Stenseth 2000). The variations in the conditions in which they must live and adapt may constrain animals to structure their behavior in both space and time (Erkert and Cramer 2006). Such flexibility may allow them to increase their reproductive success through reduced competition, predation risk, and thermoregulatory effort, as well as increased foraging efficiency (Bischof et al. 2024; Halle and Stenseth 2000). Characterizing such periodical allocations of activities in natural environments can provide critical insights into the temporal dimensions of animal ecology and inform broad ecological‐evolutionary processes that drive animal behavior.

One of the most prominent periodicities to which many organisms have adapted includes fluctuations in various environmental factors associated with day‐night cycles (Erkert and Cramer 2006; Halle and Stenseth 2000). Such factors include the recurring occurrence and duration of light and darkness periods associated with day and night length, respectively (photoperiod cycling), astronomical events (e.g., sunrise and moonrise), as well as daytime‐ and nighttime‐specific biotic and abiotic conditions (Curtis 2006; Vitaterna et al. 2001). Specifically, changes in the duration of light and darkness may trigger shifts in activity outside of a strictly diurnal (i.e., predominantly day‐active) or nocturnal (i.e., predominantly night‐active) context for certain species (Bischof et al. 2024; Donati and Borgognini‐Tarli 2006b; Hut et al. 2012; Kappeler and Erkert 2003). Such shifts can lead to “cathemerality,” which is a flexible activity pattern encompassing both daytime and nighttime periods (Tattersall 1979, 2006). Another factor that may shape animal activity patterns is the timing of various astronomical events (e.g., sunrise, sunset, moonrise, and moonset), which can influence the onset and cessation of activity (Vitaterna et al. 2001). In addition, luminosity and light intensity during the day or at night can affect an animal's ability to detect its food sources while avoiding predation and competition, thereby influencing its foraging and movement patterns. For example, some species have a peak nocturnal activity when the moon is at its brightest, relying on the brightness for better vision at night (Donati and Borgognini‐Tarli 2006a; Erkert and Cramer 2006). Alternatively, other species may avoid bright moonlight to reduce predation risk (Packer et al. 2011).

Apart from luminosity, climatic variables can also strongly shape animal activity patterns. Specifically, temperature and rainfall might influence diel activity cycling as these are often related to the length of day and night and vary seasonally. Daily changes in these variables may lead certain animal species to shift from one temporal niche to another as a physiological response, such that nocturnality may increase when temperatures are high (Curtis 2006). Understanding the extent of nocturnal activities in cathemeral species, or those predominantly active during the day, and the factors that may influence such activity levels can provide critical insights into how animals cope with the challenges associated with different environmental conditions that occur during the day and night.

Such knowledge may be particularly important for animals with significant behavioral plasticity, such as primates. While they can modify their activity patterns, they also respond significantly to the effects of human activities (de Almeida‐Rocha et al. 2017; Gaynor et al. 2018; Kalbitzer and Chapman 2018; Kifle and Bekele 2022; McLennan et al. 2017). Certain primate species may adjust their daily activities to be more active at night, allowing them to cope with the impacts of human activities and certain environmental stressors (e.g., proboscis monkeys, *Nasalis larvatus* (Kooros et al. 2022); collared brown lemurs, *Eulemur collaris* (Donati et al. 2016); chimpanzees, *Pan troglodytes schweinfurthii* (Krief et al. 2014)). Thus, characterizing primate behavioral adaptations in relation to nighttime periods can inform the dynamics of their activity patterns in the face of human‐modified environments. Additionally, as habitat transformation and degradation are increasing at an alarming rate (Estrada et al. 2017; Gardner et al. 2009), the level of light penetrating the forest canopy may also be affected, as these threats may lead to reduced canopy cover (Bischof et al. 2024; Gilbert et al. 2023). Thus, understanding how primates respond to nocturnal luminosity associated with lunar illumination at night can provide valuable insights into predicting primate responses to such threats. Moreover, as urbanization continues to expand, understanding how light influences primate activity patterns can be useful in determining the impacts of associated anthropogenic light (Beier 2006; Gilbert et al. 2023). Additionally, examining how climatic variables, such as rainfall and temperature, influence primate activity patterns can inform strategies to mitigate the effects of climate change on primate behavior (Graham et al. 2016; Kalbitzer and Chapman 2018; Korstjens and Hillyer 2016).

Here, we focused on three species of Malagasy primates—lemurs—in a tropical rainforest in Madagascar: *Eulemur rubriventer* (red‐bellied lemur), *Eulemur rufifrons* (red‐fronted lemur), and *Varecia variegata editorum* (black‐and‐white ruffed lemur). These species belong to the family Lemuridae, a monophyletic primate group endemic to the island of Madagascar (Razafindratsima et al. 2025). These highly frugivorous species live in sympatry within the rainforest of Ranomafana National Park in the southeastern part of Madagascar (Wright et al. 2011, 2012). According to a previous study on their movement patterns, *V. v. editorum* is highly mobile compared to the other two *Eulemur* spp., covering a larger home range and displaying longer daily path length (Razafindratsima et al. 2014). Previous studies have documented evidence of cathemerality in certain lemur species across four of the five genera within the Lemuridae family, including *Eulemur* spp. (Curtis and Rasmussen 2006; Donati and Borgognini‐Tarli 2006a). While *Varecia* has also been formally described as diurnal (Vasey 2005), there are anecdotal reports of nocturnal activities suggesting cathemeral behavior in wild species within this genus (e.g., Donati and Borgognini‐Tarli 2006b; Santini et al. 2015), warranting in‐depth study of their diel activity. These species also differ in various aspects of their vision. For instance, *Eulemur* spp. display dichromatic color vision and have ocular morphological traits that are intermediate between diurnal and nocturnal species, while some individual *Varecia* are trichromatic (Ankel‐Simons and Rasmussen 2008; Jacobs et al. 2019; Jacobs and Bradley 2016; Kirk 2006; Peichl et al. 2019; Tan and Li 1999). These differences may provide them with distinct adaptive advantages in the face of variable light conditions in their habitats (Jacobs et al. 2019).

We aimed to investigate the extent to which these species are active at night (i.e., nocturnal activity) and the factors that drive such activity patterns, to better understand their cathemeral behavior. We pursued the following objectives to address this goal: (1) describe their active state (i.e., when the animal is active and moves through the environment), distance traveled, and home range at night compared to during the day and with regard to daily variations in the timing of sunrise, sunset, and moonrise; (2) assess the extent to which they are cathemeral (i.e., active both during the day and at night); (3) determine what factors influence their nocturnal active state and distance traveled with regard to the length of night, nocturnal luminosity that takes into account lunar phase and illumination, and climate (temperature and rainfall). We expect the two *Eulemur* species to exhibit high levels of nocturnal activity, given that most *Eulemur* species are cathemeral (Curtis and Rasmussen 2006). We also expect *V. v. editorum* to be active at night, but not as extensively as the *Eulemur* species. We also hypothesize that all three lemur species are active at night and travel longer when the night is longer, when the moon is brighter as it is reaching its full phase, when the temperature is lower, and when there is higher rainfall. Knowledge about the diel activity patterns of these lemurs and the factors that shape their nighttime activity can help explain how they may tolerate and adapt to their changing environments.

## 2 Materials and Methods

### 2.1 Study Site

We conducted this study from May 2023 to September 2024 in the biodiverse rainforest of Ranomafana National Park (RNP) in southeastern Madagascar (21.267° S, 47.333° E), where the three studied lemur species live in sympatry. RNP features approximately 41,000 ha of montane forest, steep elevational gradients (ranging from 600 to 1500 m) (Wright and Andriamihaja 2002). Temperatures at RNP range between 4°C to 32°C (Wright et al. 2011), and precipitation varies seasonally, with an average rainfall of 3618 mm—based on a study of climatic variability between 2005 to 2016 (Dunham et al. 2018). The peak wet season typically occurs from January to March, while the peak dry season spans June to October (Dunham et al. 2011, 2018). According to the World Atlas of Artificial Night Sky Brightness (Falchi et al. 2016), RNP is subject to low artificial brightness (< 1.74), suggesting that artificial night does not influence the impacts of moonlight in this study. We collected data at the Valohoaka site, a minimally disturbed primary forest within the park where decade‐long research on numerous sympatric lemur species, including the three species focus of this study, is ongoing (Rothman et al. 2022; Wright et al. 2012).

### 2.2 Data Collections

#### 2.2.1 Lemur Activity

We captured three individual adults from three social groups per species for this study, totaling nine individuals (one female and two males per species). We affixed a radio collar equipped with GPS (E‐obs GPS‐Halsband model; https://e‐obs.de/) on each individual in May 2023. A trained wildlife veterinarian team with expertise in lemur capture in Madagascar conducted the lemur capture, administered anesthesia, and attached the collars. They immobilized the individuals using a CO_2_ air rifle to deliver 9‐mm darts containing Telazol at 10 mg/kg body weight intramuscularly. The veterinarian team also monitored heart rate, respiratory rate, and body temperature throughout the procedure. After collaring, lemurs were allowed to recover in a breathable fabric bag for approximately 3 h before being released at the exact capture site. Any individuals not fully recovered by dusk were held overnight and released the following morning. We obtained approval for animal capture, handling, and research from the University of California Berkeley's Animal Care and Use Committee (approval number AUP‐2022‐01‐15007‐1) and Madagascar's Ministry of Environment and Sustainable Development (permit number 132/23).

Using GPS collars enabled us to continuously assess their active state and movement throughout the 24‐h cycle, and to locate them for full‐day observations as part of a concurrent behavioral research project. The collars we used weighed less than 5% of the lemur's body weight. We configured each collar to record acceleration on three axes at 10‐min intervals over 24‐h periods, which we used to determine GPS recording resolution. If the acceleration event was greater than 50 cm/s (for *Eulemur* spp.) or 100 cm/s (for *V. v. editorum*), the GPS also recorded the geographic coordinates of the location every 30 min. When acceleration events were lower than these thresholds, the coordinates were recorded every 3 h. These thresholds accounted for body size and movement differences following the manufacturer's recommendation, with smaller *Eulemur* individuals (body mass: 1.82–2.54 kg; O.H. Razafindratsima unpub. data) assumed to produce weaker acceleration signals than the larger and more mobile *Varecia* (body mass: 3.95–4.25 kg; O.H. Razafindratsima unpub. data). We refer to the associated geographic coordinate points as “active points” in the analyses described below; i.e., we considered a point as “active” only if the lemur moved to a different location. Consecutive points at the same location indicate that the lemur did not move, and thus was not active for the purpose of this study. We attempted to download the data gathered by the collars once a month, as weather and detection of lemurs permitted. Due to unknown circumstances, we were unable to locate one of the collared *E. rubriventer* individuals and one *E. rufifrons* when searching for them to download data. Additionally, we were unable to download data from one of the *E. rubriventer* despite seeing it every day. Thus, our dataset comprised six lemur individuals: one male *E. rubriventer* , two males *E. rufifrons* , and three *V. v. editorum* (two males, one female). We used the daily recording as a unit of replication in our analyses to account for repeated measurements per animal. Overall, our dataset included daytime tracking for 84 days for *E. rubriventer* , 98 to 149 days for *E. rufifrons* , and 17 to 147 days for *V. v. editorum*, as well as nightime tracking for 85 nights for *E. rubriventer*, 100 to 150 nights for *E. rufifrons* , and 33 to 204 nights for *V. v. editorum* (details about our sampling efforts, including the number of days and geographic coordinate points, in Supporting Information: Table S1).

#### 2.2.2 Astronomical Data

We obtained data on the timing of sunset, sunrise, moonrise, astronomical morning twilight (i.e., before sunrise when the sun is 18° below the horizon), and astronomical evening twilight (i.e., the sun is 18° below the horizon after sunset) during our study period, from the US Naval Observatory Astronomical Application Department (https://aa.usno.navy.mil/data), using the geographic coordinates of RNP. We used these data to define the day and night periods for our study. We defined daytime as the period between the beginning of the astronomical morning twilight until the end of the astronomical evening twilight (Donati et al. 2001). Using twilight in the definition of the daytime period, rather than applying a fixed offset for sunrise and sunset, accounts for daily variations in solar geometry; also, using solely sunrise and sunset boundaries to establish day and nighttime periods may artificially inflate estimates of nocturnal activity. We conservatively incorporate both twilights into the established daytime period following the highly documented use of these low‐light periods by diurnal and cathemeral mammalian and avian taxa (e.g., Devarajan et al. 2025; Donati and Borgognini‐Tarli 2006b; Eppley et al. 2015; Kappeler and Erkert 2003). Nighttime corresponds to the remaining time within a 24‐h cycle that excludes the defined daytime. Night length ranged from 634 to 790 min.

#### 2.2.3 Lunar Luminosity

To accurately evaluate the influence of lunar luminosity, we calculated an index called Nocturnal Luminosity Index (NLI) for every 24‐h cycle. Using NLI, an above‐canopy indirect measure of lunar illumination, can avoid some of the difficulties associated with obtaining accurate measures of lunar luminosity within a rainforest setting. This approach incorporates both the fractional moon phase (the percentage of the moon's surface that is illuminated) and lunar transit times, which fluctuate periodically (Donati and Borgognini‐Tarli 2006b; Eppley et al. 2015). By considering the fraction of lunar illumination relative to both sunrise and sunset as well as the lunar disc illumination, NLI can provide a precise measure of the available lunar light (Curtis et al. 1999; Donati and Borgognini‐Tarli 2006b). We used an ad hoc program, the software Moon 2.0, as detailed in Curtis et al. (1999) and Eppley et al. (2015) to generate daily NLI values, incorporating the geographically specific sunrise, sunset, moon rise, moon set, night length, and lunar phase data. The formula to calculate NLI is as follows:

$$\mathrm{NLI}=\int_{a}^{b}\mathrm{Pdt}$$

where *a* < *b* (*dt* = 0.24 h). As Donati and Borgognini‐Tarli (2006b) stated: “When sunset precedes moonset, *a* corresponds to sunset time; when sunset precedes moonrise, *a* corresponds to moonrise time; when moonset precedes sunrise, *b* corresponds to moonset time; and when sunrise precedes moonset, *b* corresponds to sunrise time”. *P* represents the moon phase. NLI generates values ranging from 0.00 to 0.60 that are specific to the geographic coordinates of a study system at a given time period. Values closer to 0.00 correspond to periods near the new moon, when lunar illumination is minimal or absent, whereas values approaching 0.600 represent the greatest lunar illumination near the full moon phase (Eppley et al. 2015). The values of NLI in RNP during our study period ranged from 0.000 to 0.549.

#### 2.2.4 Climatic Variables

We obtained temperature and rainfall data in Ranomafana during our study period from Meteoblue historical climate database (https://www.meteoblue.com/historyplus). For the purpose of our analysis to assess the factors that influence nocturnal activity, we calculated the average temperature and total rainfall during the nighttime period for each 24‐h cycle.

### 2.3 Data Analyses

We utilized functions in R (version 4.3.2; R Core Team 2024) for data processing and analysis.

#### 2.3.1 Patterns of Active State, Distance Traveled, and Home Range

##### 2.3.1.1 Active State Patterns

We combined the timestamped geographic coordinate points from each of the radio collars, which show the animal's locations at specific times when it is active, with the astronomical data (sunrise, sunset, moonrise, and moonset) to describe the distributions of active state throughout the 24‐h cycles. Before analysis, we collapsed consecutive identical geographic coordinate points (i.e., repeated locations recorded at successive times) into one single point to avoid overrepresenting stationary periods. We calculated the frequency of the geographic coordinate points at each hour relative to the total points collected.

##### 2.3.1.2 Distance Traveled

We measured the distance traveled during the day ($DT_{d}$) and at night ($DT_{n}$) using the function *step_length* in the R‐package *amt* (Signer et al. 2019). This function measures a straight line distance between the geographic coordinates of two active points (called “step”). We projected our geographic coordinate points using the geographic projection UTM38S, which is appropriate for Madagascar (https://epsg.io/29738). We then sum up all the steps that occur during either the daytime or nighttime period to obtain the distance traveled based on points that actually occur at each time period. We performed a permutation test per lemur species to determine the statistical significance of the difference between these distances during the day vs. nighttime, using the R‐package *coin* (Hothorn et al. 2008). We considered the total distance traveled as the dependent variable and the time period (day or night) as the independent variable. We also measured a day/night ratio of hourly distance traveled by dividing the values of $DT_{d}$ per hour by $DT_{n}$ per hour. Using hourly distance instead of the total distance accounts for daily differences in the length of day and night across the study period. This ratio allows us to determine whether the lemur traveled a longer distance at night (value < 1) or not.

##### 2.3.1.3 Home Range

We estimated the spatial extent of activity for each individual lemur using the 95% Kernel Density Estimation (KDE) method with the R‐package *amt* (Signer et al. 2019) with the collected geographic coordinate points. We removed observations with fewer than five locations because this method typically requires at least five independent location points per animal. KDE provides a reliable method for showing areas of concentrated activity for each individual during specified time periods (Worton 1987). We measured the total home range across the entire study period, as well as separately for daytime and nighttime periods of each individual lemur, to obtain a more comprehensive picture of the extent of their activity.

#### 2.3.2 Cathemerality Patterns

To assess the level of cathemerality of each lemur species, we measured two metrics of active state: *mean night activity* and a *ratio of day/night activity*. To obtain the *mean night activity*, we first divided the number of active points that occurred during each nighttime period by the number of possible active points during that period. Possible points represent the number of geographic coordinate points that could have been recorded during that period if we recorded every 30 min given the setting of the collars (e.g., in an 8‐h night, there would be 16 possible points in total, one for each 30 min interval). This approach enables us to account for variations in the number of hours at each nighttime period over time. We then averaged the resulting values across our sampling period. The *day/night activity ratio* indicates the level of activity during the designated daytime period versus the nighttime period and is measured by dividing the mean number of active points during the daytime (*mean day activity*) by the mean night activity (Bray et al. 2017). We calculated the value of *mean day activity* in the same way as *mean night activity*, but with the number of recorded and possible active points that occurred during each daytime instead of nighttime. Values of the ratio approaching 1 suggest equally proportionate activity during both day and night‐time periods, values < 1 suggest increased nocturnal activity, and values > 1 indicate higher diurnal activity (Bray et al. 2017).

#### 2.3.3 Factors Influencing Nocturnal Activities

We assessed which environmental factor(s) influenced nocturnal activities in each lemur species by performing Generalized Linear Models (GLM) using the *glmmTMB* package (Brooks et al. 2017) with a *tweedie* distribution with a log link, which is well‐suited for our data with continuous and zero values. We considered the value of mean night activity as the dependent variable and NLI, average night temperature, and total night rainfall as fixed effects. Due to small sample sizes in terms of the number of individuals, we did not consider individual lemurs as a random effect. We analyzed the effect of environmental factors on distance traveled at night by each lemur species using Generalized Linear Models (GLM) with a Gamma distribution. We also considered NLI, average night temperature, and total night rainfall as predictors, and distance traveled as the dependent variable. We checked the fit of each model using functions in the *DHARMa* package (Hartig and Hartig 2017). Prior to running these models, we screened for multicollinearity among the four environmental factors (night length, NLI, average night temperature, and total night rainfall) using pairwise Pearson correlations. We considered $\left|r\right|\geq 0.7$ as indicative of strong collinearity (Gujarati and Porter 2003). All correlations were below 0.7, except for night length and temperature, which were highly correlated (*r* = −0.74). We thus ran three models and compared the Akaike Information Criterion (AIC) of the models to determine the best fit (model with the lowest AIC). The models included: (1) all four environmental factors, (2) three factors excluding night length, and (3) three factors excluding temperature. The comparison indicated that the model with temperature included but night length excluded (model #2) provided the better fit.

## 3 Results

### 3.1 Patterns of Active State, Distance Traveled, and Home Range

#### 3.1.1 Active State

**FIGURE 1:**
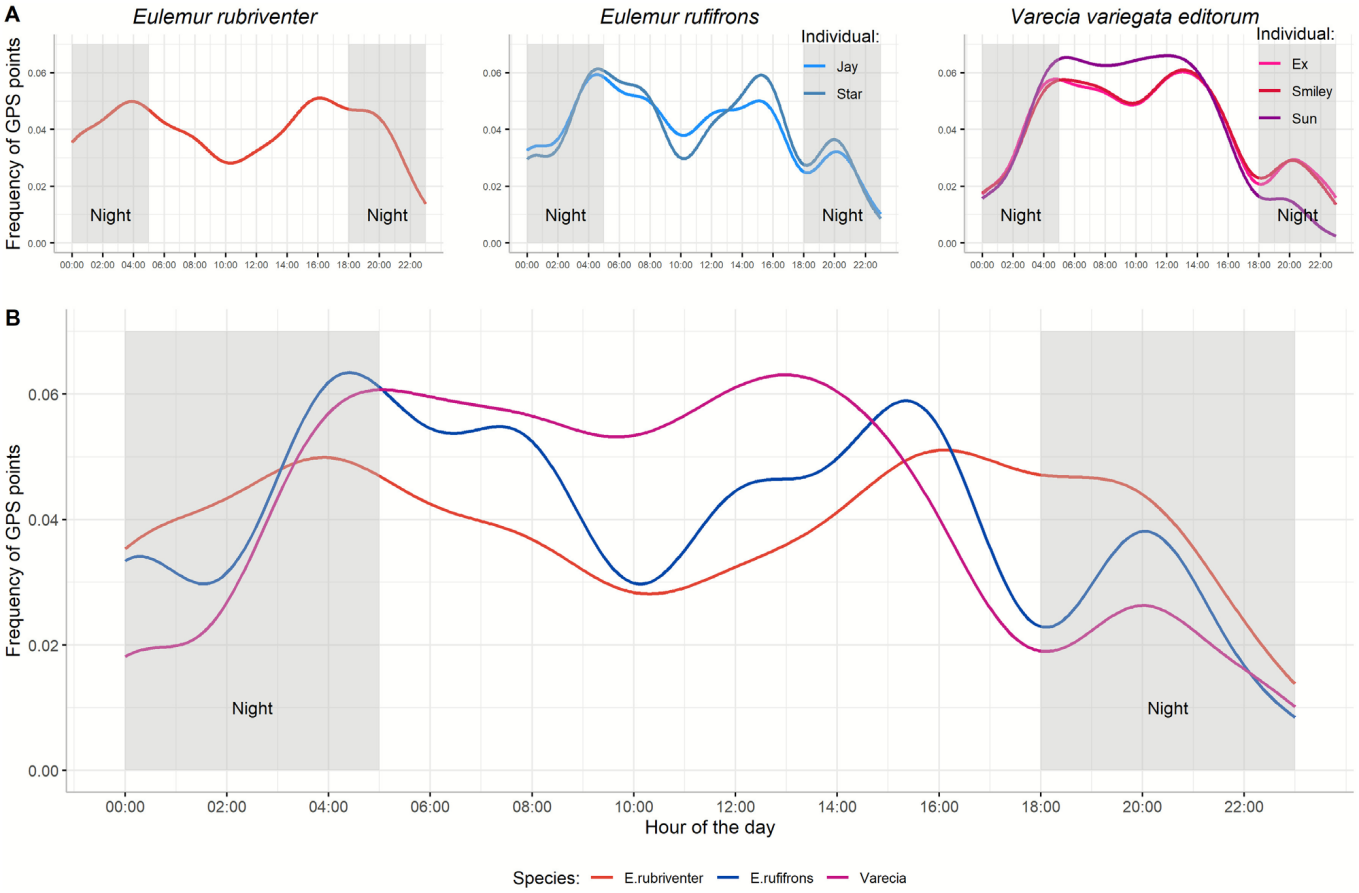
Active state during daytime and nighttime for each lemur individual (top panel) and each lemur species (bottom panel).

**FIGURE 2:**
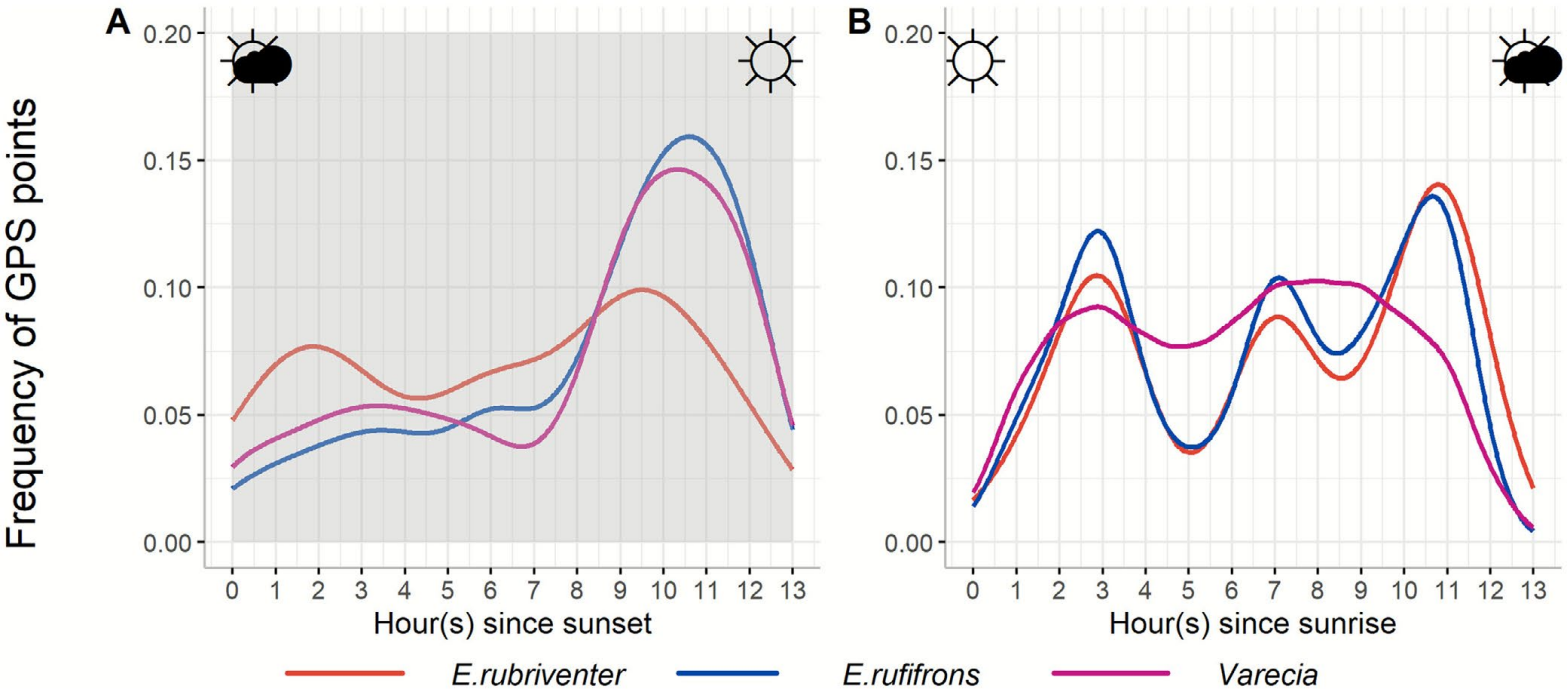
Active state according to the timing of the sunrise and sunset.

We found that all three lemur species were active at night to varying degrees (mean night activity: *E. rubriventer* = 0.08; *E. rufifrons* = 0.07–0.11; *V. v. editorum* = 0.14–0.16; Figure 1). Lemur activities even increased several hours after sunset but decreased closer to the onset of sunrise (Figure 2). The distributions of the active states for the *Eulemur* individuals slightly fluctuated throughout the day, with peaks closely tied to the morning and evening twilight periods, as indicated by the visual patterns in the frequency distribution of the geographic coordinates (Figure 1). Specifically, *E. rubriventer* exhibited a slight peak of activity between 03:00–05:00 and 15:00–18:00, with a midday dip between 09:00–11:00 (Figure 1). *Eulemur rufifrons* followed a similar pattern, with a morning activity peak from 03:30 to 5:30, a midday dip from 09:00 to 11:00, an afternoon peak between 14:00 and 16:00, followed by another evening dip between 16:00 and 19:00 before peaking again (Figure 1). *Varecia variegata editorum* exhibited a more constant activity state throughout the daytime, increasing and decreasing around the morning and evening twilight periods (Figure 1).

#### 3.1.2 Distance Traveled

**FIGURE 3:**
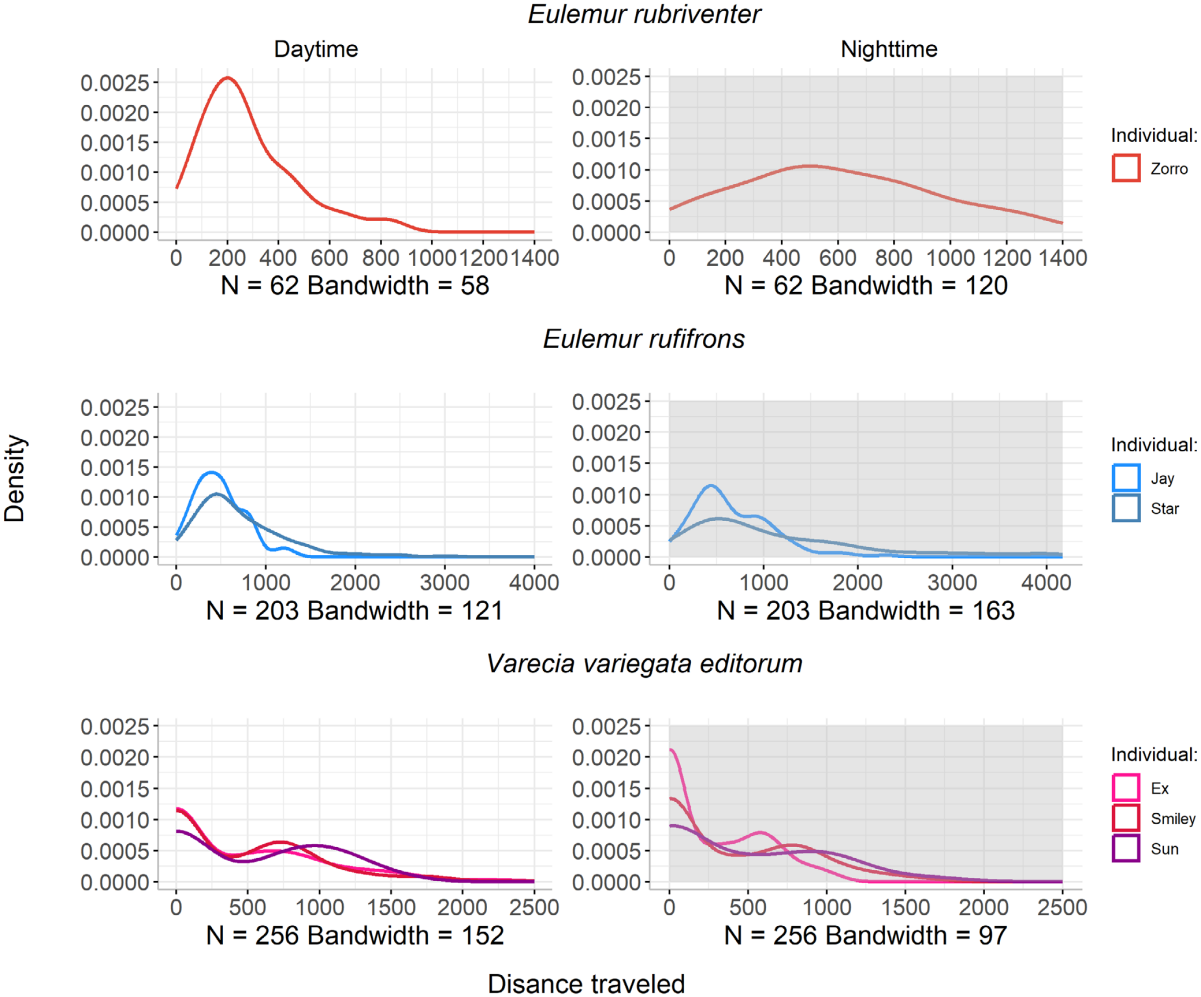
Distance traveled (m) by the three lemur species during daytime and nighttime periods.

We found that *E. rubriventer* can travel longer distances at nighttime (average: 596.37 ± 346.41; up to 1381 m in one night) than during the daytime (average: 276.95 ± 189.47 m)—such a difference was statistically significant (*t* = −6.48, *p* < 0.001; Figure 3). The day/night ratio of the hourly distance traveled for this species also indicates greater movement at night compared with the day (0.49). *Eulemur rufifrons* individuals also traveled longer at night (943.54 ± 824.45) than during the day (615.00 ± 448.74; *t* = −5.48, *p* < 0.001—Figure 3), with a maximum distance of 4171 m at night. The day/night ratio of the hourly distance traveled suggests similar patterns, indicating a greater movement during the nighttime compared to the daytime (ranging from 0.79 to 0.85). For the case of *V. v. editorum*, we found that they cover shorter distances at night (average: 329.40 ± 394.37) than during the day (average: 466.65 ± 559.45; *t* = 3.89, *p* < 0.001—Figure 3; day/night ratio ranged from 1.29 to 1.53).

#### 3.1.3 Home Range

We found that *E. rubriventer* can cover larger areas at night (32.49 ± 16.67 ha) than during the day (16.97 ± 13.58 ha). Similarly, *E. rufifrons* also had larger mean home range sizes at night (93.31 ± 139.43 ha) than during the day (52.53 ± 65.53 ha). In contrast, *V. v. editorum* individuals had a smaller home range at night (15.02 ± 13.38 ha) than during the day (17.48 ± 14.06 ha). Without considering the daily variations of home range sizes, we found that *E. rufifrons* individuals can have a much larger home range (133.07–298.92 ha) than *E. rubriventer* (36.65 ha) and *V. v. editorum* (27.06–42.44 ha).

### 3.2 Cathemerality Patterns

We determined cathemerality based on the values of day/night activity ratio, in which ratios > 1 indicate relatively more diurnal activity, ratios < 1 indicate relatively more nocturnal activity, and values near 1 indicate approximately equal activity during day and night. With a ratio of 0.64, *E. rubriventer* appears to exhibit a more nocturnal active state than the two other lemur species. *Eulemur rufifrons* shows a relatively balanced activity pattern, with activity ratios above 1 (1.34–1.37), indicating a slight preference for daytime activity periodically, but the difference between day and night activity is not as pronounced as in *V. v. editorum*, with ratios ranging from 1.88 to 2.52. These day/night ratio values for *V. v. editorum* show a predominantly diurnal behavior, even if they still exhibit nocturnal activity at times.

### 3.3 Factors Influencing Active State and Distance Traveled at Night

**FIGURE 4:**
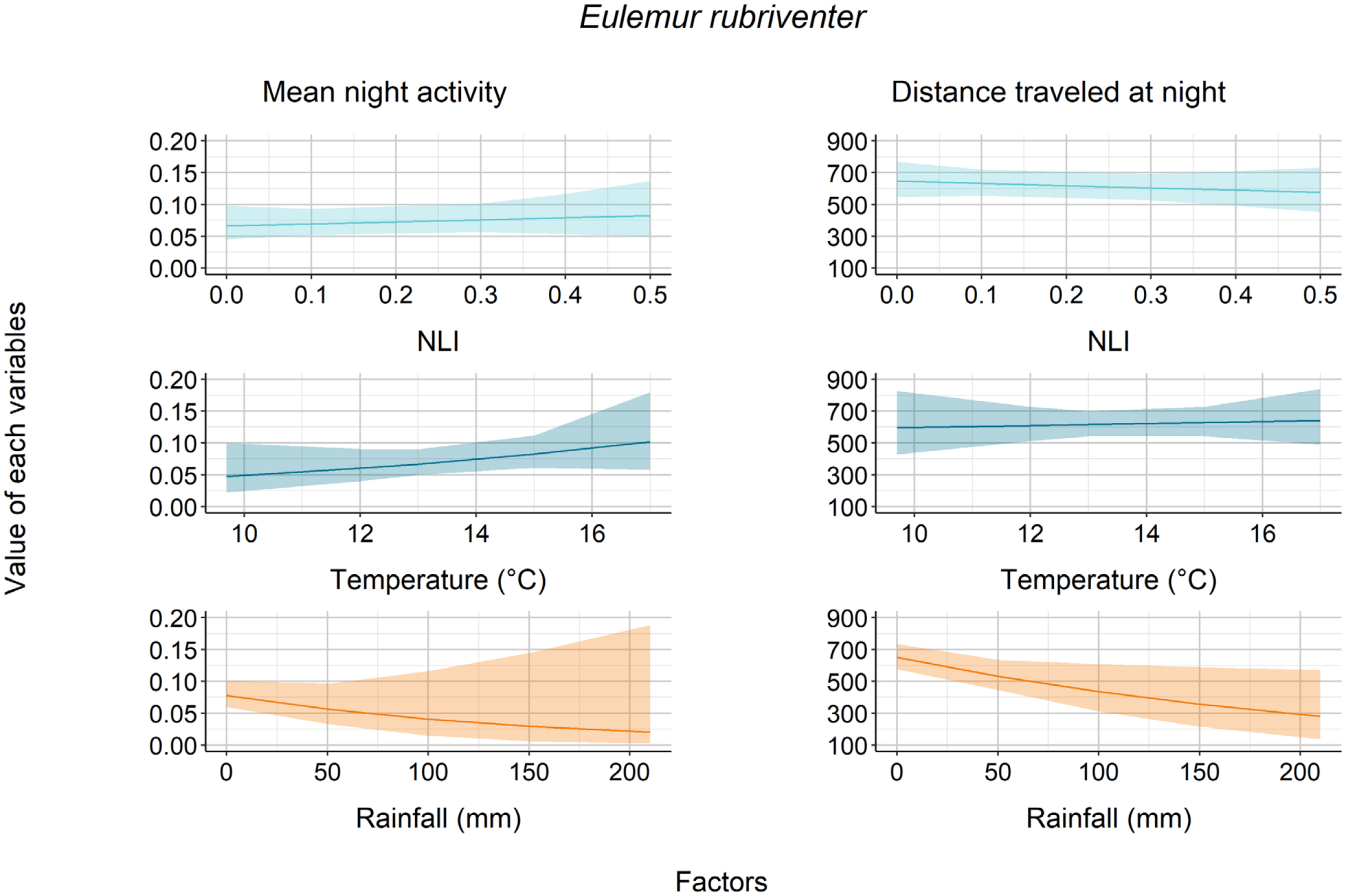
Association between each factor and the mean night activity and distance traveled for *E. rubriventer*.

**FIGURE 5:**
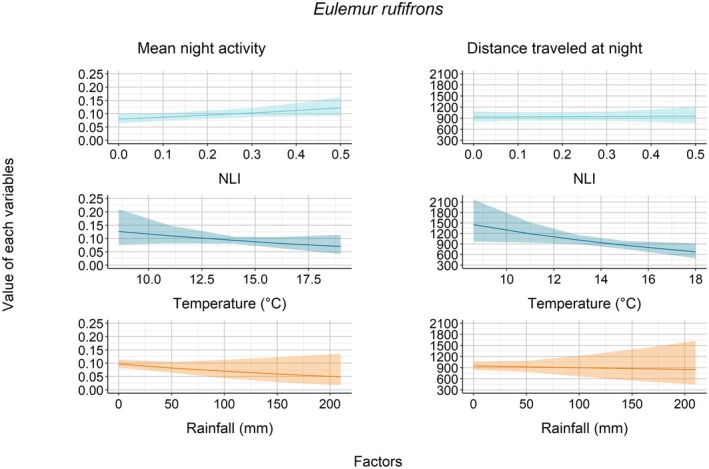
Association between each factor and the mean night activity and distance traveled for *E. rufifrons*.

**FIGURE 6:**
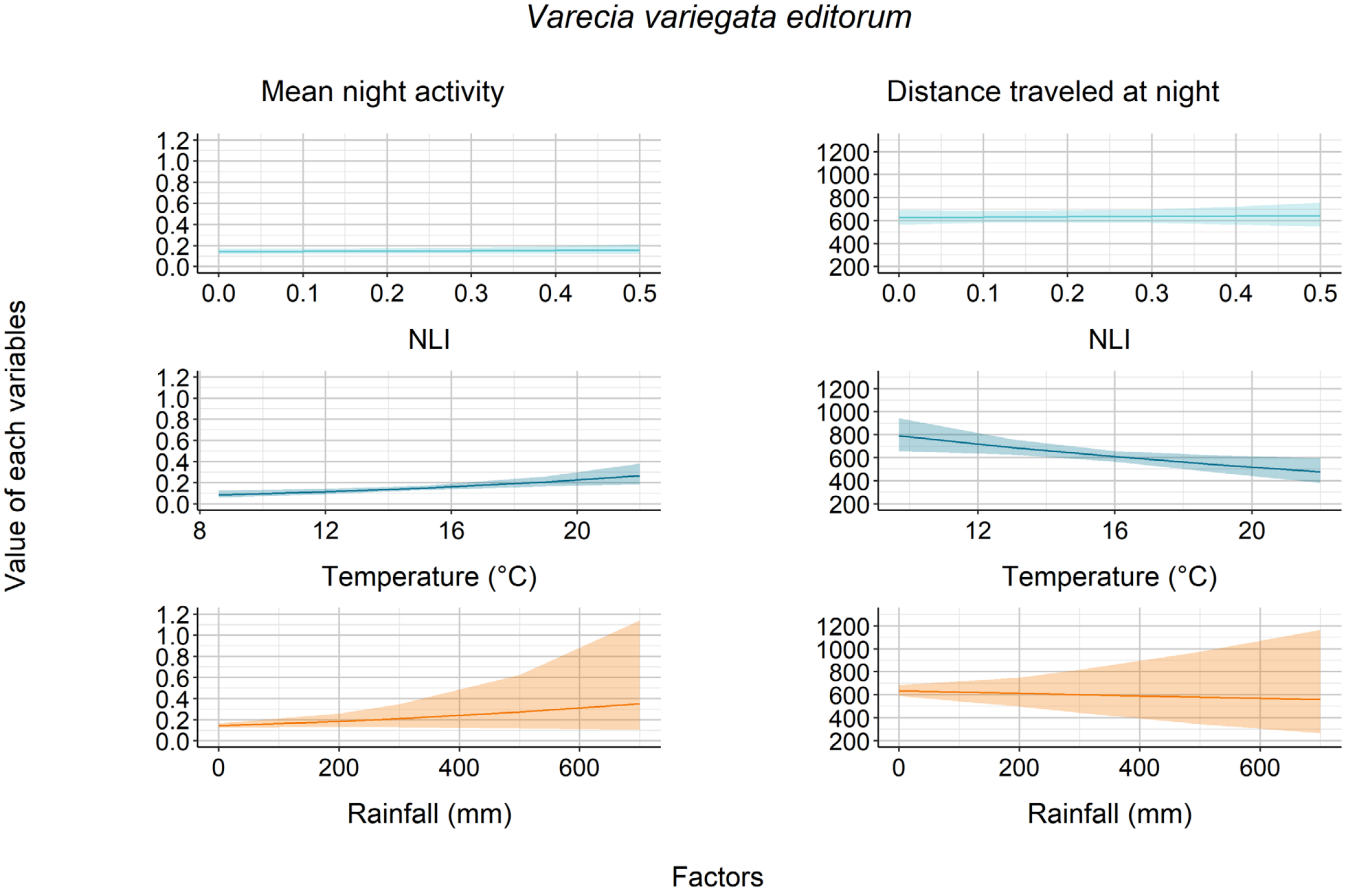
Association between each factor and the mean night activity and distance traveled for *V. v. editorum*.

For *E. rubriventer* , none of the environmental factor(s) influenced their mean night activity (NLI: *z* = 0.59, *p* = 0.55; temperature: *z* = 1.26, *p* = 0.20; rainfall: *z* = −1.17, *p* = 0.24—Figure 4). For *E. rufifrons* , NLI had a significant influence on their active state, such that they tended to be more active when NLI increased (*z* = 2.08, *p* = 0.03—Figure 5). However, temperature (*z* = −1.23, *p* = 0.22) and rainfall (*z* = −1.26, *p* = 0.21) did not have any significant effect on *E. rufifrons* 's mean night activity (Figure 5). For *V. v. editorum*, temperature influenced mean night activity (*z* = 3.18, *p* < 0.01; Figure 6); i.e., they were more active when nighttime temperatures increased. However, there was no influence of NLI (*z* = 0.58, *p* = 0.56) and rainfall (*z* = 1.44, *p* = 0.15).

Regarding the distances that each lemur traveled at night, we found a rather constant trend as the nocturnal luminosity increased, as indicated by the relationship between NLI and distance traveled ( *E. rubriventer* : *t* = −0.70, *p* = 0.49; *E. rufifrons* : *t* = 0.18, *p* = 0.86; *V. v. editorum*: *t* = 0.26, *p* = 0.80—Figures 4, 5, 6). Regarding the influence of climatic variables, we found that rainfall had a significant effect for *E. rubriventer* , i.e., they traveled longer distances when the rainfall was lower (*t* = −2.25, *p* = 0.03—Figure 4); but there was no influence of temperature (*t* = 0.26, *p* = 0.79). *Eulemur rufifrons* and *V. v. editorum* tended to travel longer distances at night when nighttime temperatures were lower ( *E. rufifrons* : *t* = −2.21, *p* = 0.03; *V. v. editorum*: *t* = −2.65, *p* < 0.01—Figures 5, 6). However, we did not find any impact of nighttime rainfall on the distance traveled at night by either *E. rufifrons* (*t* = −0.32, *p* = 0.75) or *V. v. editorum* (*t* = −0.33, *p* = 0.74).

## 4 Discussion

This study described how the active state, distance traveled, and home range of three lemur species vary during day‐ and night‐time and examined the extent to which certain environmental factors, including nocturnal luminosity, temperature and rainfall, drive their patterns of activity at nighttime. Overall, we show that all three species were active at night, to varying degrees, and under variable environmental conditions. However, species‐level interpretations should be made with caution due to limited sample sizes.

### 4.1 Nocturnal Activity Patterns in the Three Lemur Species

As expected, the three lemur species exhibited some levels of nocturnal activity. The increase in activity preceding sunrise observed in these species is consistent with what has been observed in numerous lemur species, including *Eulemur mongoz* , *Varecia variegata variegata* , *Varecia rubra* , and *Propihecus coquereli* (Bray et al. 2017; Rea et al. 2014). The distribution of activity throughout the 24‐h cycle in our study species, showing peaks pooling around sunrise and sunset, suggests the influence of astronomical cues for synchronizing diel activity, consistent with patterns observed in other lemur species in Madagascar (Kappeler and Erkert 2003; Razanaparany and Sato 2020).

The two *Eulemur* species were active as much during the day as during nighttime, suggesting cathemeral activity patterns. These findings align with previous research on other species within that genus in both captive and wild settings—e.g., *E. mongoz* (Bray et al. 2017; Curtis et al. 1999; Rasmussen 1999; Tattersall and Sussman 1975); *E. coronoatus* (Freed 1996; Wilson et al. 1989); *E. flavifrons* (Bray et al. 2017; Schwitzer et al. 2007); *E. fulvus* (Razanaparany and Sato 2020). Given that *Eulemur* species exhibit consistent cathemerality regardless of the biotic and abiotic conditions present in both wild and captive settings, they may be considered as “obligate cathemeral.” This category refers to animals that inherently display cathemerality regardless of environmental conditions (Cox and Gaston 2024).

In contrast to the two *Eulemur* species, *V. v. editorum* was more active during the day than at nighttime. While historically being classified as strictly diurnal (Vasey 2005), our findings, along with previous research on captive individuals (Bray et al. 2017) and anecdotal field observations (Donati and Borgognini‐Tarli 2006a; Wright 1999), provide compelling evidence for at least “facultative cathemerality” among *Varecia* spp. Facultative cathemerality is the ability of certain species to be active both during the day and at night due to circadian rhythms that are “weak”, i.e., obscured by one or more interacting external factors, such as seasonal food quality and abundance, thermoregulation, predator–prey relationships, interspecific competition, and lunar cycling (Botts et al. 2020; Cox and Gaston 2024; Curtis and Rasmussen 2006; Griffin et al. 2012). Madagascar's extreme climatic variability, including seasonal cyclones and fluctuating precipitation and temperature (Dunham et al. 2011, 2018), may favor facultative cathemerality in *Varecia*, as shifting environmental conditions could impose periodic demands for nighttime foraging or thermoregulatory strategies.

Cathemeral species, regardless of whether they are facultative or obligate, may have specific traits that allow them to function effectively under varying light conditions and be flexible across photoperiods. These traits include ocular adaptations that are intermediate between the visual acuity and sensitivity of strictly nocturnal or diurnal species (Kirk 2006). Nocturnal animals may possess a reflective tapetum, which is a retro‐reflective layer in the retina that increases available light under low‐light conditions (Martin and Martin 1990; Ollivier et al. 2004). Conversely, diurnal animals may possess a fovea centralis, which is a grouping of densely packed cone photoreceptors within the retina, to better generate clear, focused images (Ankel‐Simons and Rasmussen 2008; Ollivier et al. 2004; Peichl 2005). *Eulemur* and *Varecia* may have evolved distinct strategies for cathemerality and ocular morphologies that likely influence the observed differences in nocturnal activity among the species we studied. For *Eulemur* species, no evidence of a fovea centralis or a reflective tapetum has been recorded (Kirk 2006; Peichl et al. 2019), suggesting they lack morphological biases toward strictly diurnality or nocturnality. *Varecia* possesses traits characteristic of both day and night‐active primates: a fovea centralis, trichromacy (exclusively in females), and a reflective tapetum (Ankel‐Simons and Rasmussen 2008; Tan and Li 1999).

In addition to specific ocular morphology, color vision adaptation also plays a strong role in the visual ability of animals, influencing their flexibility to changes in illumination. Nocturnal animals typically have dichromatic vision, limiting their ability to see a wide range of colors, while diurnal species often exhibit trichromacy, enabling them to perceive a broader spectrum of colors, which is crucial for tasks like foraging and predator detection (Jacobs et al. 2017; Pessoa et al. 2014). In the genus *Eulemur*, individuals have only one type of opsin, making them dichromatic (Jacobs et al. 2019; Jacobs and Bradley 2016; Tan and Li 1999; Valenta et al. 2016). In *Varecia*, some individuals have trichromatic vision due to opsin gene variation, helping them detect red and green colors useful for spotting ripe fruits and young leaves in daylight (Tan and Li 1999). Even though trichromatic vision would help detect reddish foods, dichromatic with red‐shifted opsins may benefit from stronger luminance contrast, which is likely more useful in low‐light forest environments (Jacobs et al. 2019).

Another aspect of our findings is the differences in distances traveled at night compared to during the daytime. Both *Eulemur* species traveled significantly farther at night. Such a pattern suggests a strategy to maximize foraging, allowing them to locate diverse and scattered food sources throughout the habitat (Sussman and Tattersall 1976; Tarnaud 2006). It could also be due to the availability and spatial distribution of fruit resources, such that they may concentrate diurnal feeding in a particular, aggregated location when ripe fruits are abundant and increase nocturnal movement to exploit alternative food sources when fruits are scarce (Donati and Borgognini‐Tarli 2006b; Overdorff 1988). Indeed, *E. rufifrons* has been observed to depend on large fruiting trees as they often travel in large groups and frequently return to these fruiting trees (Erhart and Overdorff 2008; Razafindratsima et al. 2014). Interestingly, one male *E. rufifrons* displayed extensive movement; it traveled outside the range of the Valohoaka site, where we focused the research, but then occasionally came back, covering up to 383.37 ha of home range size in total. This behavior may be driven by certain ecological or social factors, such as searching for a mate, foraging for higher‐quality food resources, avoiding competitors, or reducing competition.

The longer nocturnal travel distances in *Eulemur* may also be used to mitigate predator risk as some of their predators in this system, such as snakes and carnivores, are active at night (Karpanty and Wright 2007). Thus, moving at night may make them less exposed to danger than if they were sleeping. Also, *E. rubriventer* may be more visible to raptors during the day because they spend more time higher in the canopy during feeding time (Overdorff 1988), which poses a higher predation risk in the daytime, forcing them to travel less. Such a predator‐avoidance strategy suggests that nocturnal foraging may be an advantageous behavior, allowing them to access food resources that would otherwise be risky to exploit during daylight hours. Furthermore, traveling longer distances at night may also allow them to reduce competition with the larger‐bodied *Varecia* that is exploiting similar food items as them (Razafindratsima et al. 2014) but travel less at nighttime, as we observed in this study.

### 4.2 Factors Influencing the Nocturnal Activity Patterns in the Three Lemur Species

As expected, we found that certain environmental factors, namely, nocturnal luminosity, temperature, and rainfall, influenced nocturnal behavior among the three lemur species, but the patterns varied. These findings corroborate other research that reported similar responses in strictly nocturnal animal species, highlighting that cathemeral species display comparable nocturnal activity patterns (Campera et al. 2022; Gursky 2003; Peterson et al. 2024). While night length was initially considered an important potential determinant of nocturnal activity, it was not included in the final models due to collinearity with temperature. However, previous studies have shown that longer nights can increase in some strictly nocturnal lemur species, such as *Lepilemur edwardsi* and *Avahi occidentalis* (Warren and Crompton 1997). Longer nights may offer an extended timeframe for completing essential activities, especially when seasonal changes delay the start of daytime activity. This delay can increase the proportion of time spent active at night (Donati and Borgognini‐Tarli 2006b), while also reducing exposure to diurnal predators (Wright et al. 1997), providing a survival advantage.

Nocturnal luminosity played a key role in shaping the nocturnal activity of *E. rufifrons* , but it was not the case for *E. rubriventer* and *V. v. editorum*. This species increased its activity by approximately 30% during periods of higher lunar illumination (Figure 5, comparing mean night activity at NLI ~0.1 vs. NLI ~0.5), indicating possible lunarphilia, i.e., positive response to moonlight (Kronfeld‐Schor et al. 2013); while *E. rubriventer* and *V. v. editorum* showed lunar neutrality (neither philia nor phobia). The lunarphilia pattern aligns with findings in other lemur species, including *E. fulvus rufus*, *E. mongoz* , *E. macaco* , *Avahi meridionalus*, and *Lemur catta* (Campera et al. 2019; Colquhoun 1998; Curtis et al. 1999; Kappeler and Erkert 2003; Parga 2011). Lunarphilia has also been observed in several nocturnal primate species in both captive settings with artificial moonlight stimulation and in the wild (Erkert and Gröber 1986; Gursky 2003; Sauther et al. 2024). The neutral response of *E. rubriventer* to nocturnal luminosity may be driven by their habitat preference at night. They may frequent the lower strata at night to reduce predation risk; thus, the amount of moonlight they receive may appear constant as the dense canopy above them can block or reduce light intensity (Curtis 2006). In *V. v. editorum*, neutral response may result from their photoperiodic flexibility, as they exhibit traits characteristic of both diurnal and nocturnal primates (Ankel‐Simons and Rasmussen 2008; Tan and Li 1999). Nevertheless, aligning activities with moonlight may be ecologically advantageous as a strategy to detect predators, reduce competitors, enhance foraging opportunities, and even exploit brighter nights for social interactions (Bischof et al. 2024; Curtis 2006; Kappeler and Erkert 2003; Kronfeld‐Schor et al. 2013; Kronfeld‐Schor and Dayan 2003). Additionally, since these species live in arboreal areas where canopy leaves may obscure their vision at night, the brighter light from the moon may help them better interact with their environment. Despite the ecological benefits of light to animal nocturnal activities, though, it may pose a risk to their survival due to increasing human‐modified environments. For instance, increased artificial light in the future may force them to be more active as a response, even if the moon is at its low illumination level. This could expose them to predators that may align their activities with moon phases. This suggests that light pollution around forest edges or nearby settlements could disrupt natural activity cycles and predator–prey dynamics. Limiting artificial lighting in or near critical habitats may, therefore, be important for maintaining natural nocturnal behaviors and reducing disturbance to wildlife.

In addition to night length and lunar illumination, the three studied lemur species also responded to climatic variables by changing their nocturnal activities with the levels of nighttime temperature and rainfall. Nighttime temperature significantly impacted *E. rubriventer* and *V. v. editorum*. They travel longer during lower temperatures, with *V. v. editorum* showing the additional pattern of increased nocturnal activity during higher temperatures. This finding is consistent with that of *E. fulvus rufus* (Donati et al. 1999; Warren and Crompton 1997) and other primate species (Campera et al. 2022; Sauther et al. 2024). The decrease in activity at the extreme end of the nighttime temperature may be a physiological response to keep energy in balance. Finally, rainfall significantly influenced *E. rubriventer* , with decreased distance traveled observed during wetter nights, i.e., they had longer distance traveled on drier nights. These findings also align with previous research in other lemur species (Curtis et al. 1999; Donati and Borgognini‐Tarli 2006b; Kappeler and Erkert 2003). These patterns highlight the importance of environmental conditions in shaping animal behavior. Warmer nighttime temperatures may reduce thermoregulatory costs, allowing individuals to increase activity and potentially maximize foraging efficiency (Riede et al. 2017). Rainfall appears to act as a limiting factor for nocturnal activity as heavy rain reduces the activity level, likely due to increased risks or energetic costs associated with moving in wet conditions (Thies et al. 2006). The behavioral flexibility observed here reflects an adaptive response to environmental variability, underscoring the potential vulnerability of these species to ongoing climate change. As temperature and rainfall patterns shift, animal activity patterns may also change. This can influence their ability to forage effectively, avoid predators, and maintain their survival.

## 5 Conclusion

This study provides new insights into the understanding of the proximate causes of lemur behavior in responses to chronobiology, showing that the nocturnal activity patterns of *Eulemur* and *Varecia* species are shaped by both circadian rhythms and environmental factors. Our findings confirm that *Eulemur* species exhibit cathemeral patterns, being active during both day and night, and demonstrate that *V. v. editorum* exhibits facultative cathemerality and primarily maintains diurnal activity with occasional nocturnal behavior under certain conditions. These activity patterns align with astronomical cues, such as the timing of sunrise and sunset, confirming the importance of light and temperature in shaping daily activity. Environmental factors, including nocturnal luminosity, also influence nocturnal behavior, with varying levels of moonlight affecting activity levels, predator detection, and foraging success. Taken together, our findings demonstrate the complex interplay among morphology, ecology, and environmental variability in shaping lemur activity patterns. Understanding these dynamics is crucial for anticipating how lemurs may respond to anthropogenic disturbances, including climate change and increasing light pollution. Future research could explore individual differences in activity patterns, as well as the effects of habitat disturbance, artificial light at night, and climate change, to provide a more complete picture of species adaptation to their environment. Overall, this study emphasizes the importance of integrating behavioral ecology with conservation strategies to support the long‐term survival of endangered species.

## Author Contributions

**Hasinavalona Rakotoarisoa:** conceptualization (equal), data curation (lead), formal analysis (equal), investigation (equal), methodology (equal), project administration (equal), supervision (equal), validation (equal), visualization (lead), writing – original draft (equal), writing – review and editing (equal). **Jadelys Tonos:** conceptualization (equal), data curation (equal), formal analysis (equal), investigation (equal), methodology (equal), supervision (equal), validation (equal), writing – original draft (supporting), writing – review and editing (equal). **Hannah Hilden‐Reid:** formal analysis (supporting), writing – original draft (equal), writing – review and editing (equal). **Onja H. Razafindratsima:** conceptualization (equal), formal analysis (equal), funding acquisition (lead), investigation (equal), methodology (equal), project administration (equal), resources (lead), supervision (equal), writing – original draft (lead), writing – review and editing (equal).

## Funding

This work was supported by the University of California Berkeley; Hellman Fellowship; Regents Junior Faculty Fellowship.

## Conflicts of Interest

The authors declare no conflicts of interest.

## Supporting information


**Data S1:** ece372819‐sup‐0001‐supinfo.pdf.

## Data Availability

All the required data are available in Dryat at https://doi.org/10.5061/dryad.905qftv0m.
