# Supplementary material for: Living in the Dark: Exploring the Factors Driving Nocturnal Activity in Three Lemur Species
Source: Ecol Evol. 2026 Feb 19;16(2):e72819. doi: 10.1002/ece3.72819 (PMC12917331; doi:10.1002/ece3.72819)
Supplement: Supplementary file 1 — Data S1: ece372819‐sup‐0001‐supinfo.pdf. [file ECE3-16-e72819-s001.pdf]

Supplementary Information

Table S1.

Number of GPS points collected for each lemur individual. Values in parentheses show the number of days of data collection per lemur individual.

Species	*Eulemur rubriventer*	*Eulemur rufifrons*		*Varecia variegata editorum*		
Individual name	Zorro	Star	Jay	Smiley	Sun	Ex
daytime	344 (84)	1065 (149)	550 (98)	1306 (147)	306 (17)	1860 (105)
nighttime	415 (85)	914 (150)	490 (100)	880 (77)	163 (33)	1151 (204)
